# Association between Sour Taste SNP *KCNJ2*-rs236514, Diet Quality and Mild Cognitive Impairment in an Elderly Cohort

**DOI:** 10.3390/nu13030719

**Published:** 2021-02-24

**Authors:** Celeste Ferraris, Alexandria Turner, Christopher Scarlett, Martin Veysey, Mark Lucock, Tamara Bucher, Emma L. Beckett

**Affiliations:** 1 School of Environmental and Life Sciences, The University of Newcastle, Ourimbah, NSW 2258, Australia; alexandria.turner@uon.edu.au (A.T.); c.scarlett@newcastle.edu.au (C.S.); mark.lucock@newcastle.edu.au (M.L.); tamara.bucher@newcastle.edu.au (T.B.); emma.beckett@newcastle.edu.au (E.L.B.); 2 School of Medicine & Public Health, The University of Newcastle, Gosford, NSW 2250, Australia; martin.veysey@newcastle.edu.au; 3 Hull York Medical School, University of Hull, Cottingham HU6 7RX, UK; 4 Priority Research Centre for Physical Activity and Nutrition, The University of Newcastle, Callaghan, NSW 2308, Australia; 5 Hunter Medical Research Institute, Callaghan, NSW 2308, Australia

**Keywords:** sour, taste, genetics, *KCNJ2* gene, rs236514, polymorphism, SNP, cognitive impairment, dementia, elderly, diet

## Abstract

Differences in sour-taste thresholds have been identified in cognition-related diseases. Diet is a modulator of cognitive health, and taste perception influences dietary preferences and habits. Heritable genetics and polymorphisms in the *KCNJ2* gene involved in the transduction of sour taste have been linked to variations in sour taste and non-gustatory functions. However, relationships between sour taste genetics, mild cognitive impairment, and diet quality are yet to be elucidated. This study investigated the associations between the presence of the *KCNJ2*-rs236514 variant (A) allele, diet quality indices, and mild cognitive impairment evaluated by the Mini-Mental State Examination (MMSE), in a secondary cross-sectional analysis of data from the Retirement Health & Lifestyle Study. Data from 524 elderly Australians (≥65y) were analyzed, using standard least squares regression and nominal logistic regression modeling, with demographic adjustments applied. Results showed that the presence of the *KCNJ2*-A allele is associated with increased proportions of participants scoring in the range indicative of mild or more severe cognitive impairment (MMSE score of ≤26) in the total cohort, and males. These associations remained statistically significant after adjusting for age, sex, and diet quality indices. The absence of association between the *KCNJ2*-A allele and cognitive impairment in women may be related to their higher diet quality scores in all indices. The potential link between sour taste genotype and cognitive impairment scores may be due to both oral and extra-oral functions of sour taste receptors. Further studies are required on the role and relationship of neurotransmitters, sour taste genotypes and sour taste receptors in the brain, and dietary implications, to identify potential risk groups or avenues for therapeutic or prophylactic interventions.

## 1. Introduction

As the population ages, cognition-related disease prevalence in Australia and the directly associated costs are expected to rise by 90% over the next twenty years [1]. Worldwide, a 300% increase in dementia prevalence is expected by 2050 [2]. The significant impact on quality of life [3,4] and the relationship to increased risk of concurrent chronic diseases [5] highlights an immediate need for novel management strategies and broader understandings of the risk factors associated with the onset of cognitive decline. 

Diet is a known modulator of cognitive health, [6,7,8] and is established as a preventative tool in cognition-related diseases [6,9]. Dietary preferences are strongly influenced by perception of the five key tastes—bitter, sweet, sour, salt, and umami [10,11]. Sour taste is stimulated by acids in foods [12] such as berries, citrus fruits, and fermented foods [13]. These sour foods are commonly consumed in diets found to be effective in the prevention of cognitive decline [6,7,8]. The relationships between differences in taste perception and intake of cognitive-health-promoting foods is an under-researched area, particularly for sour taste. 

Recent research has demonstrated loss of taste function in mild cognitive impairment (MCI), dementia [14,15], and Alzheimer’s disease (AD) [15,16,17]. Specifically, for sour taste, sour-threshold testing has revealed that higher concentrations are required before sourness is detected by patients with dementia and AD [14,16]. Taste decline in cognitive impairment has been found to be independent of other influencing factors, such as prescription drugs, salivation, zinc levels [15], and ageing [17,18]. However, the direction and consequences of the associations have yet to be established. Therefore, the more direct relationships between sour taste and cognition require further investigation. 

Sour-taste thresholds and perception are modulated by receptor genetics. Early research has shown heritable genetics significantly contribute to variations in sour recognition thresholds [19], perceived pleasantness and intensity of sour taste, and the frequency with which sour foods are consumed [20]. Single-nucleotide polymorphisms (SNPs) in taste receptors have been associated with alterations in taste, including sour perception [10]. Taste-receptor SNPs that increase intensity of taste perception have been shown to reduce liking of tastants [21,22]. The *KCNJ2* (Potassium Inwardly Rectifying Channel Subfamily J Member 2) gene is found in the Type III sour-sensing taste cells [23] and is linked to the magnitude of sour-taste transduction [24]. One study found that carriers of the *KCNJ2*-rs236514 variant allele (A) had a higher preference (liking) for sour [25], which may suggest that the SNP reduces transduction. Whether the SNP and reduced sensitivity to sour are related to risk for cognition-related diseases has yet to be elucidated. 

Therefore, the possible relationship between *KCNJ2*-rs236514 sour genotype, diet quality, and a marker of cognitive impairment was assessed in a well-characterized elderly cohort. This cross-sectional analysis used scores from the Mini-Mental State Examination (MMSE) as an index for cognitive impairment and assessed the relationship between this index and the common sour-taste SNP, *KCNJ2*-rs236514, and three diet-quality indices. 

## 2. Materials and Methods

### 2.1. Subjects

Data for this secondary analysis were obtained from a cross-sectional cohort of elderly subjects (≥65 years) from the Retirement Health and Lifestyle Study (RHLS) [26,27,28]. Randomly selected participants lived independently or in retirement villages in the local Gosford and Wyong state government areas of the Central Coast region of NSW, Australia. There were no exclusion criteria based on pre-existing health conditions, cognitive or otherwise; however, participants were required to have sufficient language and cognition skills to be able to provide written informed consent. From the 831 participants that took part in the initial study, only those who provided blood samples and valid food-frequency questionnaires (FFQ) were genotyped for *KCNJ2*-rs236514 and were hence included in the current study (n = 524). The Human Research Ethics Committee of the University of Newcastle granted ethics approval (Reference No. H-2008-0431) [26,27,28]. 

### 2.2. Demographics and Anthropometrics

Interviewer-administered questionnaires collected data on age, sex, education, income, and smoking history [26,27,28]. Body Mass Index (BMI = weight (kg)/height (m^2^)) was calculated by using height and weight measures obtained by following the International Society for the Advancement of Kinanthropometry (ISAK) guidelines [29]. Height was recorded to the nearest 0.01 cm, using the stretch stature method. Weight was recorded to the nearest 0.01 kg on digital scales (Wedderburn© UWPM150 Platform Scale).

### 2.3. Genotyping

Whole fasted blood samples were collected into EDTA-lined, tubes and DNA were isolated from peripheral blood cells [28,30] following the manufacturer’s instructions (QIAGEN QIAmp DNA mini-kit) [31]. Both the blood samples and DNA were stored at −20 °C [28,30]. TaqMan assay (Applied Biosystems, ThermoFisher Scientific, Waltham, MA, USA 02451) was used to genotype the *KCNJ2*-rs236514 allelic variants, according to the manufacturer’s protocols. 

### 2.4. Cognitive Assessment

Cognitive function was assessed with the MMSE, a valid and reliable tool widely used and recommended in research [32,33]. The MMSE measures orientation, registration, attention, calculation, memory, recall, and drawing ability to diagnose dementia [34]. The threshold of ≤26 was used to classify those with MCI, inclusive of those with more severe forms of cognitive impairment. [35,36,37,38,39]. 

### 2.5. Diet Quality Indices

Dietary data were obtained by a previously validated FFQ [40]. Foodworks^TM^ (V.2.10.146) software was used to analyze participants’ intake of the 225 food items [41]. An FFQ was deemed invalid if data were incomplete or if energy intake reports were >30,000 kJ/d or <3000 kJ/d. 

Three diet-quality indices were generated from the FFQ data. The Dietary Guideline Index (DGI) is a 150-point index based on Australian dietary and alcohol intake guidelines [42,43] and national indicators of food and nutrition [44]. The DGI provides a measure of diet quality that reflects dietary diversity, key nutrient intake from core food groups, and healthful food (e.g., vegetables) and unhealthy food intakes [26,45]. The Australian Recommended Food Score (ARFS) is a 74-point index based on the Australian Dietary Guidelines that focuses on variety as the key indicator of diet quality [26,46]. Finally, the Australian Healthy Eating Index (Aust-HEI) is a 60-point index that considers variety, adherence to healthy food-choice guidelines; higher consumption of fruits, vegetables, and low-fat milk; and lower consumption of meat, saturated fats, and low-nutrient-density foods to be a diet of higher quality [26,47].

### 2.6. Statistical Analysis

Data were analyzed, using JMP (Pro V.14.2.0; SAS Institute Inc., Cary, NC, USA 27513). Continuous variable distributions (means, 95% confidence intervals and standard deviations) and categorical variable distributions (number and percentage of cohort) describe the cohort characteristics. Where categories had insufficient numbers of participants for statistical analysis, groups were consolidated for further analyses. Ex-smokers and current smokers were collapsed into a single “history of smoking” category; income was collapsed into two categories (≤$20,000 and >AUD $20,000 per year), and education categories were ≤Trade qualification and TAFE (Technical and Further Education) or higher. As previous research has demonstrated a potential sex dimorphism in sour-taste perception, with women finding sour more intense and having higher sensitivity to sourness [48,49,50], analyses were stratified by sex. 

*KCNJ2*-rs236154 allele frequency was reported as number and percentage of the study cohort and analyzed by presence or absence of the *KCNJ2* variant allele (A). MMSE scores were categorized nominally based on the defined thresholds (≤26 = MCI; >26 = normal cognition). Statistical significance of continuous variables was examined through standard least squares regression and for categorical variables through nominal logistic regression (χ^2^, *p*-values, odds ratios, and 95% confidence intervals). Analyses were adjusted for age, sex, education, income, smoking status, and BMI. As diet is a contributing factor to cognitive health [6,7], and diet is modified by taste perception, further analyses with adjustments for the diet quality indices were made. The *p*-values are presented to the first significant figure, and threshold *p*-values of <0.05 were considered statistically significant. 

## 3. Results

### 3.1. Participant Characteristics

The ages of the 524 participants ranged from 65 to 94 years (mean 77.6 years, SD ± 6.7) (Table 1). The mean BMI was 28.6 kg/m^2^ (Table 1). Age and BMI did not vary by sex. The mean diet-quality scores were 96.8/150 points (DGI), 29.0/74 points (ARFS), and 30.3/60 points (Aust-HEI) (Table 1). Females had higher mean diet quality scores than males across all three diet quality indices (DGI: *p* = 0.0005, ARFS: *p* = 0.003 and Aust-HEI: *p* = <0.0001, Table 1).

The cohort was 54.4% female (Table 2). Most participants reported earning > AUD $20,000 per year and being educated at TAFE level or higher (Table 2). Men were more likely than women to have higher incomes (85.6% vs. 53.8%, *p* = <0.0001), to be educated at TAFE level or higher (75.7% vs. 60.2%, *p* = 0.0001), and to have a history of smoking (66.5% vs. 35.1%, *p* = <0.0001) (Table 2). 

### 3.2. Genotype Distributions

The frequency of the *KCNJ2*-rs236514 variant allele (A) was 0.56, and the ancestral allele (G) was 0.44. A large proportion of the participants (81.1%) carried the *KCNJ2*-A allele (AA or AG genotypes), and there were no differences by sex (Table 3). 

### 3.3. MMSE Distributions

MMSE scores indicative of MCI occurred in 17.6% of the cohort, 16.5% of females, and 18.8% of males (Table 4). There were no statistically significant differences by sex.

### 3.4. Relationships between Presence of the KCNJ2-rs236514 Variant (A) Allele and Confounding Variables

The presence of the *KCNJ2*-rs236514 variant (A) allele was more likely in older females (*p* = 0.04) and was associated with higher BMI in males (*p* = 0.002) (Table 5). There were no differences in the presence of the *KCNJ2*-A allele in the distributions of sex, income, education, and smoking (*x*^2^ range = 0.7–2.5, all *p*-values ≥ 0.05).

### 3.5. Relationships between MMSE Scores and Confounding Variables

In the total cohort and males, MMSE scores indicative of MCI were associated with lower diet-quality scores (ARFS) (*p* = 0.002, *p* = 0.0004 respectively) (Table 6). Older women were more likely to have MMSE scores indicative of MCI than younger women (*p* = 0.009) (Table 6). Lower education was associated with an increased likelihood of meeting the MMSE threshold score indicative of MCI in the total cohort, and in women (*p* = 0.04, *p* = 0.02 respectively) (Table 7).

### 3.6. Relationships between KCNJ2-rs236514 and MCI (MMSE) 

Those with the *KCNJ2*-A allele were more likely to have MMSE scores indicative of MCI in the unadjusted (*p* = 0.03) and age and sex-adjusted models (*p* = 0.04), but not in the fully adjusted model (*p* = 0.09) (Table 8). After stratifying by sex, male *KCNJ2*-A allele carriers were more likely to meet the MMSE threshold scores for MCI than non-carriers, in the unadjusted (*p* = 0.02) and age-adjusted (*p* = 0.02) models (Table 9). There were no relationships found between MCI (MMSE) and the categorical variables amongst women.

#### 3.7. Relationships between KCNJ2-rs236514 and MMSE Scores Indicative of MCI, Adjusting for the Diet Quality Indices

As scores on the ARFS index were associated with MCI (MMSE) (Table 6), further analyses were conducted adjusting for the diet-quality indices (Table 10 and Table 11). The relationship between the presence of the *KCNJ2*-A allele and MMSE scores indicative of MCI remained significant after adjusting for each of the diet-quality indices in the total cohort (DGI: *p* = 0.04; ARFS: *p* = 0.03; and Aust-HEI: *p* = 0.03; Table 10), and in men (DGI: *p* = 0.02; ARFS: *p* = 0.007; and Aust-HEI: *p* = 0.02; Table 11).

## 4. Discussion

This is the first study to investigate the relationships between sour-taste genetics and cognitive impairment. The findings demonstrate that the presence of the *KCNJ2*-rs236514 A allele, a variant associated with altered mRNA stability and protein expression of the taste receptor [51,52,53], increases the likelihood of MMSE scores indicative of MCI in the total cohort and men. Diet quality was not a confounding factor in these relationships. However, the absence of association between female *KCNJ2*-A allele carriers and MCI (MMSE) may be related to their higher diet-quality scores in all indices. 

While there are limitations to a cross-sectional study design, as a first step, these findings provide new directions for research that may inform new management strategies for cognition-related diseases. Integrating these findings with previous related studies on the biology and function of the SNP, taste genetics, and sour taste informs the hypothesis that the *KCNJ2*-A allele may be reducing overall signal transduction [10,19,20,21,22,23,24]. In the absence of directly comparable research, results are contextualized by related studies on the *KCNJ2* gene, rs236514 SNP, and the structural and biological commonalities in taste, the brain, and cognitive impairment. 

Three previous studies have demonstrated expression and dysregulation in taste receptors in the brain of AD [54], schizophrenia [55], and Parkinson’s [56] patients that is not reflective of neuronal loss alone. Sour taste is altered in cognition-related diseases with detection and recognition of sour decreasing and taste thresholds increasing in dementia and AD [14,16]. Our results suggest that the *KCNJ2*-rs236514 variant allele may have a role in the cognitive impairment that characterizes these conditions, particularly in light of the SNP’s demonstrated impact on sour taste [25]. Furthermore, the decreased sensitivity to sour perception in dementia and AD supports the hypothesis that the SNP may be reducing sour-taste transduction. 

Sour-taste transduction involves the release of neurotransmitters commonly altered in cognition-related diseases. Sour compounds stimulate Type III taste cells to release 5-hydroxytryptophan (5-HT), gamma-aminobutyric acid (GABA), and norepinephrine (NE) [57]. Lower levels of GABA [58,59], NE [60], and 5-HT [61,62], as well as reduced 5-HT neurotransmission [63], are found in the brain of cognitively impaired individuals. *KCNJ2* genes are highly expressed in the same areas of the brain (cerebral cortex, amygdala, thalamus, hippocampus, and basal ganglia) [64,65] that demonstrate changes in cognitive decline [66,67,68,69]. If as hypothesized the *KCNJ2*-A allele is reducing transduction, the release of 5-HT, GABA, and NE from the extra-oral taste receptors in the brain may be impacted. While these studies indirectly link sour taste, neurotransmitters, and cognitive impairment, direct research in this area has not been undertaken. The possible extra-oral functions of the gene and SNP require further investigation.

Men carrying the *KCNJ2*-A allele were more likely to meet the MMSE threshold scores for MCI than those not carrying the allele. The singular study available on sour taste and the *KCNJ2*-rs236514 SNP did not analyze data by sex [10], and neither did the studies demonstrating decreased sensitivity to sour perception in cognition-related diseases [14,16]. However, adjustments for sex were made in all studies, and no significant effects were found [10,14,16]. Sex dimorphisms in sour-taste perception have been demonstrated previously in two research projects. Women found sour to be more intense [49,50] and had a higher preference for sourness [50]. The reasons for sex-based differences in sour taste, and in the relationship between the *KCNJ2*-rs236514 SNP and MCI in our study, are unknown and require further investigation. 

In addition to the cross-sectional design of the study, further limitations should be considered. The absence of research for direct comparative analysis means that the findings are contextualized by associated studies and hypotheses. Both genetics and cognition-related diseases are multi-factorial. However, the availability of data on relevant potential confounders in this study allowed for adjustments for common associated factors to be made. In addition to the cohort being well-characterized, the sample size was large, the sex distribution was even, and 81.3% of participants carried the *KCNJ2*-A allele. Even though the results may not be generalizable to wider age groups, the age of participants (≥65 years) is relevant to the higher and increasing prevalence of cognition-related diseases in the elderly. Furthermore, the significant relationships between carriage of the *KCNJ2*-A allele and MCI (MMSE) existed independently of adjustments for age, broadening the scope for application in other age groups. 

## 5. Conclusions

Mild-to-severe cognitive impairment (MMSE) was found to be more likely in the presence of the *KCNJ2*-rs236514 variant (A) allele in this elderly Australian cohort. In the context of previous knowledge correlating altered sour-taste perception to cognition-related diseases, this novel study indicates a more fixed genetic link. While not conclusive, this pilot study on a convenient cross-sectional sample suggests that further research is warranted. Studies on relationships between neurotransmitters common to both sour taste and cognition-related disease, extra-oral functions of sour taste receptors particularly in the brain, the SNP’s influence on direction and magnitude of transduction, and the role of diet quality are needed to address the gaps in the body of knowledge. 

## Figures and Tables

**Table 1:** Distribution of continuous variables by total cohort and by sex.

Variable	Total			Females			Males			*p*
	Mean (SD)	Min	Max	Mean (SD)	Min	Max	Mean (SD)	Min	Max	
Age (years)	77.6 (±6.7)	65.0	94.0	77.7 (±6.7)	65	94	77.4 (±6.8)	65	93	0.6
BMI (kg/m^2^)	28.6 (±4.8)	17.1	46.3	28.6 (±5.0)	17.6	46.3	28.6 (±4.5)	17.1	45.4	0.9
DGI	96.8 (±15.9)	30.9	132.6	99.0 (±16.3)	30.9	132.6	94.2 (±15.0)	51.8	130.4	0.0005
ARFS	29.0 (±8.0)	6.0	50.0	29.9 (±8.1)	6	50	27.8 (±7.7)	10	49	0.003
Aust-HEI	30.3 (±9.5)	4.9	50.8	32.0 (±9.1)	6.4	50.8	28.3 (±9.7)	4.9	46.5	<0.0001

SD, standard deviation; BMI, Body Mass Index; DGI, Dietary Guideline Index (150 points); ARFS, Australian Recommended Food Score (74 points); Aust-HEI, Australian Health Eating Index (60 points).

**Table 2:** Distribution of categorical variables by total cohort and by sex.

Variable	Total	Females	Males	*p*
	*n* (%)	*n* (%)	*n* (%)	
**Sex**				
Males	239 (45.6)			
Females	285 (54.4)			
**Income**				<0.0001
≤AUD $20,000 per year	161 (31.5)	127 (46.2)	34 (14.4)	
>AUD $20,000 per year	350 (68.5)	148 (53.8)	202 (85.6)	
**Education**				0.0001
≤Trade qualification	171 (32.7)	113 (39.8)	58 (24.3)	
TAFE or higher	352 (67.3)	171 (60.2)	181 (75.7)	
**Smoking**				<0.0001
History of smoking	259 (49.4)	100 (35.1)	159 (66.5)	
Never smoked	265 (50.6)	185 (64.9)	80 (33.5)	

TAFE, Technical and Further Education.

**Table 3:** *KCNJ2*-rs236514 variant (A) allele distributions by total cohort and by sex.

Genotype	Total	Females	Males	*p*
	*n* (%)	*n* (%)	*n* (%)	
*KCNJ2*-A allele present	425 (81.1)	236 (82.8)	189 (79.0)	0.3
*KCNJ2*-A allele absent	99 (18.9)	49 (17.2)	50 (20.9)	

**Table 4:** *MMSE* distributions by total cohort and by sex.

MMSE	Total	Female	Male	*p*
	*n* (%)	*n* (%)	*n* (%)	
MCI (≤26)	92 (17.6)	47 (16.5)	45 (18.8)	0.5
Normal cognition (>27)	432 (82.4)	238 (83.5)	194 (81.2)	

MMSE, Mini-Mental State Examination; MCI, mild cognitive impairment.

**Table 5:** Demographic and clinical characteristics by presence of the *KCNJ2*-rs236514 variant (A) allele in the total cohort and by sex (continuous variables).

Variable	Total			Females			Males		
	LSM (95% CoI)		*p*	LSM (95% CoI)		*p*	LSM (95% CoI)		*p*
	A Allele Present	A Allele Absent		A Allele Present	A Allele Absent		A Allele Present	A Allele Absent	
Age (years)	77.8 (77.2–78.5)	76.5 (75.1–77.8)	0.07	78.1 (77.2–78.9)	75.9 (73.2–78.9)	0.04	77.5 (76.5–78.5)	77.0 (75.1–78.9)	0.7
BMI (kg/m^2^)	28.7 (28.2–29.2)	28.0 (27.0–28.9)	0.2	28.4 (27.7–29.1)	29.1 (27.6–30.5)	0.4	29.0 (28.4–28.1)	26.8 (25.6–28.1)	0.002
DGI	97.2 (95.7–98.7)	94.9 (91.8–98.0)	0.2	99.7 (93.1–102.3)	97.7 (93.2–101.3)	0.5	94.7 (92.5–96.8)	92.2 (88.0–96.4)	0.3
ARFS	28.9 (28.1–29.7)	29.1 (27.5–30.7)	0.9	29.5 (28.5–30.5)	31.9 (29.7–34.2)	0.06	28.2 (24.2–28.4)	26.3 (24.2–28.4)	0.1
Aust-HEI	30.5 (29.6–31.4)	29.5 (27.7–31.4)	0.4	32.0 (30.8–33.2)	32.2 (29.6–34.7)	0.9	28.7 (27.3–30.0)	27.0 (24.3–29.7)	0.3

LSM, least squares mean; CoI, confidence interval; BMI, Body Mass Index; DGI, Dietary Guideline Index (150 points); ARFS, Australian Recommended Food Score (74 points); Aust-HEI, Australian Health Eating Index (60 points).

**Table 6:** Demographic and clinical characteristics by MMSE scores indicative of MCI in the total cohort and by sex (continuous variables).

Variable	Total			Females			Males		
	MMSE LSM (95% CoI)		*p*	MMSE LSM (95% CoI)		*p*	MMSE LSM (95% CoI)		*p*
	≤26	>27		≤26	>27		≤26	>27	
Age (years)	78.4 (77.0–79.8)	77.4 (76.7–78.0)	0.2	80.1 (78.1–82.0)	77.2 (76.4–78.1)	0.009	76.7 (74.7–78.8)	77.6 (76.6–78.5)	0.5
BMI (kg/m^2^)	28.7 (27.6–29.7)	28.5 (28.1–29.0)	0.8	28.4 (26.8–30.0)	28.6 (27.9–29.2)	0.8	28.9 (27.5–30.3)	28.5 (27.8–29.1)	0.6
DGI	94.7 (91.4–98.1)	97.2 (95.7–98.7)	0.2	97.3 (92.5–102.1)	99.3 (97.2–101.3)	0.5	92.1 (87.5–96.6)	94.6 (92.5–96.7)	0.3
ARFS	26.6 (24.9–28.2)	29.4 (28.7–30.2)	0.002	29.0 (26.6–31.4)	30.1 (29.1–31.1)	0.4	24.1 (21.8–26.3)	28.6 (27.6–29.7)	0.0004
Aust-HEI	29.4 (27.4–31.4)	30.5 (29.6–31.4)	0.3	32.6 (30.0–35.2)	31.9 (30.8–33.1)	0.7	26.1 (23.2–29.0)	28.8 (27.4–30.2)	0.1

MMSE, Mini-Mental State Examination; LSM, least squares mean; CoI, confidence interval; BMI, Body Mass Index; DGI, Dietary Guideline Index (150 points); ARFS, Australian Recommended Food Score (74 points); Aust-HEI, Australian Health Eating Index (60 points).

**Table 7:** Demographic characteristics by MMSE scores indicative of MCI in the total cohort and by sex (categorical variables).

Variable	Total			Females			Males		
	MMSE LSM (95% CoI)		*p*	MMSE LSM (95% CoI)		*p*	MMSE LSM (95% CoI)		*p*
	≤26	>27		≤26	>27		≤26	>27	
Sex	0.8 (0.5–1.2)	1.3 (0.8–2.0)	0.3						
Income	0.8 (0.5–1.3)	1.3 (0.8–2.1)	0.3	0.8 (0.4–1.4)	1.3 (0.7–2.4)	0.4	1.0 (0.4–2.4)	1.0 (0.4–2.6)	0.9
Education	0.6 (0.4–0.9)	1.6 (1.0–2.6)	0.04	0.5 (0.3–0.9)	2.2 (1.2–4.1)	0.02	0.8 (0.4–1.7)	1.3 (0.6–2.7)	0.5
Smoking	0.7 (0.5–1.1)	1.4 (0.9–2.2)	0.1	1.0 (0.5–1.9)	1.0 (0.5–1.9)	1.0	0.5 (0.2–1.0)	1.0 (1.0–4.2)	0.05

MMSE, Mini-Mental State Examination; LSM, least squares mean; CoI, confidence interval.

**Table 8:** Odds of MMSE scores indicative of MCI by *KCNJ2*-A allele presence in the total cohort, in unadjusted and adjusted models.

	Unadjusted		Model 1		Model 2	
	*x*^2^ (*p*)	OR (95% CoI)	*x*^2^ (*p*)	OR (95% CoI)	*x*^2^ (*p*)	OR (95% CoI)
MMSE ≤26	4.3 (0.03)	2.0 (1.0–4.0)	4.1 (0.04)	2.0 (1.0–4.0)	2.8 (0.09)	1.8 (1.0–3.7)

MMSE, Mini-Mental State Examination; OR, odds ratio; CoI, confidence interval; Model 1, adjusted for age and sex; Model 2, adjusted for age, sex, income, education, smoking, and BMI.

**Table 9:** Odds of MMSE scores indicative of MCI by *KCNJ2*-A allele presence in unadjusted and adjusted models, by sex.

	Females						Males					
	Unadjusted		Model 1		Model 2		Unadjusted		Model 1		Model 2	
	*x*^2^ (*p*)	OR (95% CoI)	*x*^2^ (*p*)	OR (95% CoI)	*x*^2^ (*p*)	OR (95% CoI)	*x*^2^ (*p*)	OR (95% CoI)	*x*^2^ (*p*)	OR (95% CoI)	*x*^2^ (*p*)	OR (95% CoI)
MMSE ≤26	0.6 (0.4)	1.4 (0.6–3.6)	0.2 (0.7)	1.2 (0.5–3.1)	0.1 (0.7)	1.2 (0.5–3.1)	5.0 (0.02)	3.0 (1.0–8.8)	5.1 (0.02)	3.0 (1.0–8.9)	3.4 (0.06)	2.7 (0.9–8.2)

MMSE, Mini-Mental State Examination; OR, odds ratio; CoI, confidence interval; Model 1, adjusted for age; Model 2, adjusted for age, income, education, smoking, and BMI.

**Table 10:** Odds of MMSE scores indicative of MCI by *KCNJ2*-A allele presence in the total cohort, in models adjusting for diet quality.

	Model 1		Model 2		Model 3	
	*x*^2^ (*p*)	OR (95% CoI)	*x*^2^ (*p*)	OR (95% CoI)	*x*^2^ (*p*)	OR (95% CoI)
MMSE ≤26	4.3 (0.04)	2.0 (1.0–4.0)	4.5 (0.03)	2.0 (1.0–4.1)	4.5 (0.03)	2.0 (1.0–4.1)

MMSE, Mini-Mental State Examination; OR, odds ratio; CoI, confidence interval; Model 1, adjusted for DGI (Dietary Guideline Index); Model 2, adjusted for ARFS (Australian Recommended Food Score); Model 3, adjusted for Aust-HEI (Australian Health Eating Index).

**Table 11:** Odds of MMSE scores indicative of MCI by *KCNJ2*-A allele presence in models adjusting for diet quality, by sex.

	Females						Males					
	Model 1		Model 2		Model 3		Model 1		Model 2		Model 3	
	*x*^2^ (*p*)	OR (95% CoI)	*x*^2^ (*p*)	OR (95% CoI)	*x*^2^ (*p*)	OR (95% CoI)	*x*^2^ (*p*)	OR (95% CoI)	*x*^2^ (*p*)	OR (95% CoI)	*x*^2^ (*p*)	OR (95% CoI)
MMSE ≤26	0.5 (0.5)	1.4 (0.6–3.5)	0.5 (0.5)	1.4 (0.5–3.5)	0.6 (0.4)	1.4 (0.6–3.6)	5.0 (0.02)	3.0 (1.0–9.0)	7.4 (0.007)	3.9 (1.3–12.0)	5.6 (0.02)	3.2 (1.1–9.6)

MMSE, Mini-Mental State Examination; OR, odds ratio; CoI, confidence interval; Model 1, adjusted for DGI (Dietary Guideline Index); Model 2, adjusted for ARFS (Australian Recommended Food Score); Model 3, adjusted for Aust-HEI (Australian Health Eating Index).

## Data Availability

Where ethically appropriate, data can be accessed by contacting the corresponding author.
